# Report of an Isolated L5 Radiculopathy Caused by an L2-3 Disc Herniation and Review of the Literature

**DOI:** 10.7759/cureus.2552

**Published:** 2018-04-30

**Authors:** Alan A Stein, Frank Vrionis, Patricio S Espinosa, Shaye Moskowitz

**Affiliations:** 1 Boca Raton Regional Hospital, Marcus Neuroscience Institute, Boca Raton, USA; 2 Neurological Surgery, Marcus Neuroscience Institute, Boca Raton, USA; 3 Neurology, Marcus Neuroscience Institute, Boca Raton Regional Hospital, Boca Raton, USA; 4 Neurological Surgery, UC Health, Cincinatti, USA

**Keywords:** lumbar disc herniation, neurosurgery, spine surgery, lumbar radiculopathy, monoradiculopathy

## Abstract

Intervertebral disc herniation is a common cause of radiculopathy. Disc herniations occurring in the lumbar spine typically affect the nerve root exiting under the pedicle of the vertebral body, one level caudal. However, in rare cases, a disc herniation can cause remote isolated radicular symptoms. The authors present the case of a 70-year-old male who presented with an acute, new-onset, left-sided foot drop, low back pain, and a classic L5 monoradiculopathy. Imaging revealed a large, left-sided paracentral extruded L2-3 disc and the absence of any pathology at the L4/5 level. Although the patient’s clinical presentation and imaging did not classically correlate, it was felt that the L2-L3 disc was the etiology of the patient’s L5 radiculopathy and a left L2-3 microsurgical discectomy was performed. At the six-week follow-up, his foot drop was near normal, sensation was intact with minimal paresthesias, and he remained pain-free. At the one-year follow-up, he experienced full resolution of his foot drop and remained symptom-free. Although rare, disc herniations may cause isolated, remote, painful mononeuropathies not related to the direct level of nerve root compression and should be considered along with other etiologies of peripheral neuropathies.

## Introduction

Intervertebral disc herniation is a common cause of radiculopathy. Disc herniations occurring in the lumbar spine typically affect the nerve root exiting under the pedicle of the vertebral body, one level caudal. However, in rare cases, a disc herniation can cause remote isolated radicular symptoms. We describe a rare case of lumbar five (L5) nerve root monoradiculopathy resulting from an intervertebral disc herniation between the second and third lumbar vertebral levels (L2-3). We also reviewed the literature regarding similar cases and summarize those findings in this report.

## Case presentation

A seventy-year-old male, with atrial fibrillation and remote prostate cancer, presented with a left-sided foot drop, which had developed three weeks prior. He presented complaining of low back pain and radicular symptoms prominent in the left fifth lumbar (L5) nerve root territory, with pain in the anterolateral aspect of the distal leg and numbness along the dorsum of his foot. The physical examination was significant for a significant weakness in the left tibialis posterior, extensor hallucis longus, and anterior tibialis, presenting as a foot drop and a weakness in foot inversion. All other muscles were normal in strength, particularly the quadriceps, hip adductors, iliopsoas, and tibialis posterior. Sensory findings included decreased pin-prick detection in the L5 dermatome. Osteotendinous patellar and Achilles tendon reflexes were normal and present bilaterally. A spine exam was notable for normal alignment and range of motion with no spinal or paraspinal point tenderness. He had a markedly positive ipsilateral straight-leg raise test at under thirty degrees, no pain with hip or knee passive range of motion, no trochanteric sensitivity, and no Tinel’s sign at the fibular head.

**Figure 1:**
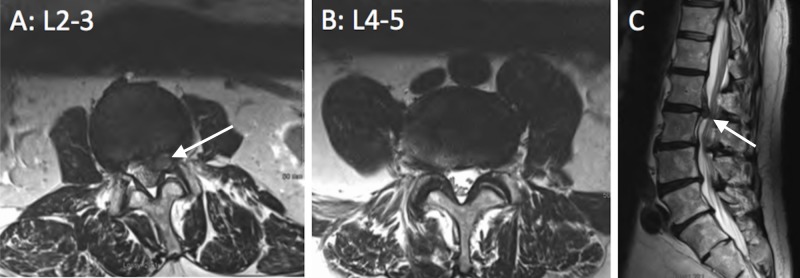
Lumbar Spine MRI 1A. L2-3 disc, axial T2 sequence through the L2-3 disc space showing a large left disc herniation with lateral recess and foraminal stenosis. 1B. L4-5 disc, axial T2 sequence through the L4-5 disc space showing minimal arthritic changes and no impingement of the exiting and passing roots. 1C. Sagittal T2 sequence slightly to the left of midline showing a large L2-3 disc herniation and only minor changes elsewhere. The intradural roots at the L4/5 level can be clearly visualized and are non-edematous and non-displaced. MRI: magnetic resonance imaging

Magnetic resonance imaging (MRI) findings revealed a large, left-sided paracentral extruded L2-3 disc with lateral recess and foraminal stenosis and mass effect on the ventral aspect of the thecal sac (Figures 1A-1B). Other disc levels showed degenerative changes including minor lateral recess stenosis at L3-4 and, notably, a normal L4-5 disc (Figure 1C). Serology was unremarkable. Although the patient’s clinical presentation and imaging did not classically correlate, it was felt that the L2-L3 disc was the etiology of the patient’s L5 radiculopathy, and a left L2-3 microsurgical discectomy was performed. There were no complications postoperatively, and he had immediate pain relief. At the six-week follow-up, his foot drop was near normal, sensation was intact with minimal paresthesias, and he remained pain-free. At the one-year follow-up, he experienced full resolution of his foot drop and has regained full function and sensation and remains pain-free.

## Discussion

The most classic presentation of lumbar disc herniation with nerve root compression is radicular pain in the lower extremity following dermatomal distribution. Symptoms of L5 nerve root compression typically afflict the anterior tibialis, extensor hallucis longus, extensor digitorum brevis, and lateral gastrocnemius muscles, as they receive their primary motor innervation via the L5 nerve root. An L2-3 disc herniation classically presents with pain and sensory changes from the anterior thigh medially to the knee. The myotomal distribution typically includes the quadriceps femoris and hip adductors but may also include the iliopsoas. In this patient, there were only L5 clinical symptoms, with no clinical findings that corresponded to the third lumbar nerve root. A sufficiently large disc herniation could cause a polyradiculopathy but would include the primary level and components of caudal levels if the passing nerves were compressed within the thecal sac as well. This patient is unusual in that only the fifth lumbar dermatome and myotome was affected, and L3 and L4 were not.

A monoradiculopathy resulting from a remote lumbar disc herniation is a very rare finding. In the published literature, only one case of L2-L3 disc herniation, three cases of L1-L2 disc herniation, and one case of L1-L2 and/or L2-L3 stenosis have been associated with L5 radiculopathy [1-5]. We describe these cases in Table 1.

**Table 1:** Cases Reported in the Literature

Author	Nerve Root	Description
Hidalgo-Ovejero, AM et al. [1]	L5 nerve root	Degenerative stenosis of the L1-L2 and L2-L3 level
Hidalgo-Ovejero, AM et al. [3]	L5 nerve root	L2-L3 disc herniation
Korovessis, P et al. [4]	L5 nerve root	L1-L2 disc herniation
Yasuda, Muneyoshi et al. [2]	L5 nerve root	L1-L2 disc herniation
Shirado, O et al. [5]	L5 nerve root	L1-L2 disc herniation

We hypothesized in our case that the L5 nerve root was compressed at the L2-3 lumbar level, as the L5 nerve root travels down the spinal canal as part of the cauda equina. Within the thecal sac, the more distal sacral nerve roots are situated dorsally, whereas the lumbar nerve roots course ventrally within the thecal sac prior to exiting the spinal canal via the designated intervertebral foramina. Sufficient laxity in the sacral and contralateral roots allowed medial displacement by the herniation and sufficient space lateral to the herniation to allow the exiting of the third lumbar nerve and the passing of the intradural fourth lumbar nerve roots. Yasuda et al. described a similar case of L5 radiculopathy caused by a disc herniation at L1-2. In his case, it was postulated that the disc herniation resulted in venous congestion and selective L5 root compression [2]. In our case, the L2-3 disc herniation was found to be the only compressive lesion. Further studies are needed for the comprehensive understanding of the pathophysiology of the selective L5 nerve root monoradiculopathy in the setting of L2-3 stenosis.

Alternative painful mononeuropathies should be considered in this patient given the discordant imaging. A nerve conduction study and electromyography were considered, though were not obtained, as his clinical syndrome was of an isolated L5 radiculopathy with pain and sensorimotor findings, and they were not likely to alter the management of the large herniation. Furthermore, we chose to urgently treat him given the acute foot drop, rather than delay surgery. Entrapment syndromes, particularly of the common peroneal nerve, can cause a foot drop. The symptoms are often slowly progressive rather than acute. The loss of ankle inversion in our patient is a reflection of weakness in the tibialis posterior muscle, innervated by the posterior tibial nerve. As the posterior tibial nerve does not pass by the fibular head and the common peroneal nerve, along the distribution of the L5 myotome, the involvement of this nerve is indicative of a spinal etiology rather than the easily confused peroneal nerve entrapment syndrome [6]. Diabetic nerve complications are often insidious polyneuropathies, with stocking/glove sensory symptoms, depressed reflexes, and not clinically applicable in this patient [7]. Infectious etiologies include Lyme disease and HIV [8], but they are rarely mononeuropathies and were not clinically likely without the history or evidence of exposure. Vasculitides may present with a painful neuropathy affecting the vasa nervorum [9-10]. Such a presentation would be exceedingly rare to have no additional constitutional symptoms from a systemic inflammatory reaction, normal serology, rapid onset, and isolated to a single nerve, though it is a theoretic possibility. Nutritional deficits, polyarteritis nodosa, lupus, and sarcoidosis may produce mononeuropathies, and our patient did not exhibit any related signs of a systemic condition to consider these.

## Conclusions

Rare disc herniations may cause isolated, remote painful mononeuropathies and should be considered along with other diagnoses. Surgical management may be appropriate for such patients in a manner similar to the presentations of clinical syndromes with imaging-concordant spinal pathology.

## References

[1] Hidalgo-Ovejero AM, Garcia-Mata S, Martinez-Grande M, Maravi-Petri E, Izco-Cabezon T (1998). L5 root compression caused by degenerative spinal stenosis of the L1-L2 and L2-L3 spaces. Spine (Phila Pa 1976).

[2] Yasuda M, Nakura T, Kamiya T, Takayasu M (2011). Motor evoked potential study suggesting L5 radiculopathy caused by L1-2 disc herniation: case report. Neurologia Medico-Chirurgica.

[3] Hidalgo-Ovejero AM, Garcia-Mata S, Sanchez-Villares JJ, Lasanta P, Izco-Cabezon T, Martinez-Grande M (2003). L5 root compression resulting from an L2-L3 disc herniation. Am J Orthop (Belle Mead NJ).

[4] Korovessis P, Baikousis A, Stamatakis M, Katonis P (1998). Monoradiculopathy of the fifth lumbar nerve root due to lumbar disc herniation between lumbar one and lumbar two vertebrae. J Spinal Disord.

[5] Shirado O, Matsukawa S, Kaneda K (1996). Herniation of the disc between the first and second lumbar vertebrae with a monoradiculopathy of the fifth lumbar nerve root. J Bone Joint Surg.

[6] Iwamoto N, Kim K, Isu T, Chiba Y, Morimoto D, Isobe M (2016). Repetitive plantar flexion test as an adjunct tool for the diagnosis of common peroneal nerve entrapment neuropathy. World Neurosurg.

[7] Pasnoor M, Dimachkie MM, Barohn RJ (2013). Diabetic neuropathy part 2: proximal and asymmetric phenotypes. Neurol Clin.

[8] Somer T, Finegold SM (1995). Vasculitides associated with infections, immunization, and antimicrobial drugs. Clin Infect Dis.

[9] Collins MP, Arnold WD, Kissel JT (2013). The neuropathies of vasculitis. Neurol Clin.

[10] Gwathmey KG, Burns TM, Collins MP, Dyck PJ (2014). Vasculitic neuropathies. Lancet Neurol.

